# The Evidence for Geary’s Theory on the Role of Mitochondrial Functioning in Human Intelligence Is Not Entirely Convincing

**DOI:** 10.3390/jintelligence8030029

**Published:** 2020-07-20

**Authors:** Anna-Lena Schubert, Dirk Hagemann

**Affiliations:** Institute of Psychology, Heidelberg University, Hauptstr. 47-51, 69117 Heidelberg, Germany; dirk.hagemann@psychologie.uni-heidelberg.de

**Keywords:** general intelligence, mitochondrial functioning, behavioral genetics, cognitive enhancement, cognitive processes

## Abstract

Geary (2018, 2019) suggested that heritable and environmentally caused differences in mitochondrial functioning affect the integrity and efficiency of neurons and supporting glia cells and may thus contribute to individual differences in higher-order cognitive functioning and physical health. In our comment, we want to pose three questions aimed at different aspects of Geary’s theory that critically evaluate his theory in the light of evidence from neurocognitive, cognitive enhancement, and behavioral genetics research. We question (1) if Geary’s theory explains why certain cognitive processes show a stronger age-related decline than others; (2) if intervention studies in healthy younger adults support the claim that variation in mitochondrial functioning underlies variation in human intelligence; and (3) if predictions arising from the matrilineal heredity of mitochondrial DNA are supported by behavioral genetics research. We come to the conclusion that there are likely many more biological and social factors contributing to variation in human intelligence than mitochondrial functioning.

## 1. Introduction

In the summary of his theory on intelligence differences, Geary (2019) outlined the elementary ideas of his hypothesis that individual differences in mitochondrial functioning are the main driving force for individual differences in human intelligence (Geary 2018, 2019). Geary suggested that heritable and environmentally caused differences in mitochondrial functioning affect the integrity and efficiency of neurons and supporting glia cells and may thus contribute to individual differences in higher-order cognitive functioning and physical health. His theory aimed to account for different phenomena of intelligence research, including the positive manifold (Spearman 1904), the association between intelligence and health (Batty et al. 2007; Deary 2008; Der et al. 2009)^1^, the joint age-related decline in performance across different cognitive domains (Rhemtulla and Tucker-Drob 2011; Salthouse 2009; Salthouse and Ferrer-Caja 2003; Tucker-Drob 2011; Tucker-Drob et al. 2014), and the greater variability in intelligence test scores in males than in females (Hedges and Nowell 1995; Johnson et al. 2008; Wai et al. 2010). As such, Geary’s theory is very compelling because it provides an elegant account of many important empirical phenomena of intelligence research. Moreover, it integrates biological and cognitive approaches to studying intelligence by acknowledging that elementary cognitive processes underlying human intelligence are bound to the integrity of the brain, which is one of many bodily organs and, therefore, affected by basic physiology. 

In our comment, we want to pose three questions aimed at different aspects of Geary’s theory that critically evaluate his theory in the light of evidence from neurocognitive, cognitive enhancement, and behavioral genetics research. 

### 1.1. Can Geary’s Theory Explain Why Certain Cognitive Processes Show a Stronger Age-Related Decline Than Others?

Geary (2018) argued that parameters of certain cognitive processes such as working memory (WM), which “refers to a system, or set of processes, holding mental representations temporarily available for use in thought and action” (Oberauer et al. 2018, p. 886), are more strongly related to intelligence and show greater age-related decline than parameters of other cognitive processes, such as short-term memory (STM), due to higher energy demands. Because more energy-demanding systems show greater metabolic activity than less energy-demanding systems, they create more reactive oxygen, which results in more rapid accumulation of mutations across the life span.

This argument relies on the assumption that working memory processes require intermodular neural connections that are supported by intact axonal connections between modules, whereas other cognitive processes rely to a larger degree on intramodular connections. Hence, both higher energy use and the dependence on a larger number of intermodular axonal connections are supposed to account for the greater age-related decline in WM than in STM. We believe that this conclusion is neither supported by cognitive theories on memory systems nor by neuroscientific evidence. Cognitive psychologists have been abandoning a strict distinction between WM and STM towards a broader definition of working memory that includes short-term memory processes (Oberauer et al. 2018). If there is a conceptual distinction between the two memory systems, it typically entails that STM involves the mere maintenance of information, whereas WM involves the simultaneous maintenance and manipulation of information (Cowan 2017). This additional manipulation of information may indeed require higher levels of cellular energy. However, there is ample evidence that the encoding, maintenance, and retrieval of information required both in WM and STM rely on long-range intermodular neural connections. In particular, these processes have been shown to require the integration of neural activity elicited from the ventrolateral prefrontal cortex, the inferior temporal cortex, the ventral posterior parietal cortex, and the medial temporal lobe (Nee and Jonides 2011, 2013a, 2013b; Öztekin et al. 2008). Therefore, it is implausible to assume that individual differences in axonal integrity and neural plasticity or age-related myelin degradation account for greater age-related decline in WM than in STM, as both WM and STM processes require long-distance intermodular information transmission.

In addition, language-related abilities such as verbal understanding and verbal production, which are reflected in measures of crystallized intelligence, stay relatively stable across the lifespan and show a much more decelerated age-related decline than both WM and STM (Horn and Cattell 1967; Salthouse 2004). However, the same processes are known to heavily rely on intermodular connections (Barbey 2018; Silbert et al. 2014). As such, the discrepancy in age-related trajectories of fluid and crystallized intelligence also contradicts the idea that networks relying on long-distance intermodular information transmission show a greater age-related decline due to more extensive myelin degradation.

### 1.2. Do Intervention Studies in Healthy Younger Adults Support the Claim That Variation in Mitochondrial Functioning Underlies Variation in Human Intelligence?

One very elegant test of Geary’s hypothesis would be to increase mitochondrial functioning through pharmaceutical or training-based interventions and to assess whether these interventions show effects on general cognitive performance. Geary (2018) cited several studies in which nutritional supplementation using ketone agents, creatine, coenzyme Q10, or resveratrol, which affect different aspects of mitochondrial functioning, led to increases in cognitive performance in older adults and in adults with neurodegenerative diseases, such as Parkinson’s and Alzheimer’s disease (Henderson et al. 2009; Li et al. 2015; Witte et al. 2014). However, these effects seem to only hold for older individuals or individuals with certain neurodegenerative diseases, while evidence for cognitive enhancement by nutritional supplementation in younger adults is limited. 

A recent review on the beneficial effects of creatine supplementation summarized three studies assessing the effects on intelligence test performance in younger adults (Avgerinos et al. 2018). Creatine supplies energy to cells with increased energy demands, and its higher-energy phosphate bonds can be used for immediate ATP replenishment in energy-demanding situations, such as cognitive tasks, that require high neural energy use (Persky and Brazeau 2001). However, the studies summarized by Avgerinos et al. (2018) do not provide compelling evidence that increased creatine availability increases cognitive functioning in healthy young adults. One of these studies found no effect of creatine supplementation on cognitive performance (Rawson et al. 2008), while another found a large effect in comparison to a placebo condition that could, however, be attributed to substantial baseline differences in intelligence between the two groups (Ling et al. 2009). The only study that reported compelling evidence for enhanced cognitive performance following creatine supplementation contained a sample of vegetarians (Rae et al. 2003), who typically show lower plasma creatine levels due to their diet (Delanghe et al. 1989). 

Similarly, effects of resveratrol administration seem to protect against neurodegenerative effects on cognitive function (Foti Cuzzola et al. 2011; Sun et al. 2010; Witte et al. 2014). Resveratrol is best known as one of several wine polyphenols thought to be responsible for the health benefits of moderate regular wine consumption (Keylor et al. 2015). It is synthesized by plants undergoing infectious or ionizing radiation and acts as a potent antioxidant (Malhotra et al. 2015; Salehi et al. 2018). However, despite showing promising effects in the treatment of neurodegenerative diseases, several studies failed to observe any benefits of resveratrol administration on cognitive functioning in healthy young adults despite observing resveratrol-related increases in cerebral blood flow (Kennedy et al. 2010; Wightman et al. 2014, 2015; Wong et al. 2013). 

Taken together, these results suggest that these nutritional supplementations only affect cognitive functioning in older adults, in individuals with certain neurodegenerative diseases, and in individuals with dietary restrictions. In a similar vein, animal studies have shown that experimental interventions modulating mitochondrial fusion/fission dynamics can facilitate neural regeneration (Chien et al. 2018), underlining the role of mitochondrial dynamics in degenerative neurocognitive processes. Hence, these studies cannot be cited to support Geary (2018) hypothesis that individual differences in mitochondrial functioning underlie natural variation in intelligence; instead, they only lend credence to the idea that age-related cognitive decline may be mediated by impairments in mitochondrial functioning.

### 1.3. Are Predictions Arising from the Matrilineal Heredity of Mitochondrial DNA Supported by Behavioral Genetics Research?

There is evidence that variations in mitochondrial DNA (mtDNA) have an effect on mitochondrial functioning and several body systems, including the brain (see Geary 2018, p. 1034). When, in turn, differences in mitochondrial functioning have an effect on general intelligence, then one would expect that differences in mtDNA are also associated with differences in intelligence. In particular, individuals with similar mtDNA would be expected to show rather similar levels of intelligence, and individuals with dissimilar mtDNA would be expected to show rather dissimilar levels of intelligence. Because mtDNA has a matrilineal heredity, it follows that the mtDNA of a mother and her offspring is much more similar (identical devoid of mutations) than in the case of a father and his offspring. In short, one may conclude that the mother–offspring correlation of intelligence must be larger than the father–offspring correlation of intelligence and that the size of this difference gives a clue on the magnitude of how much mtDNA differences would drive differences in intelligence. However, empirical data do not support this prediction. Whitley et al. (2011) analyzed data from the 1958 cohort of the National Child Development Study, including offspring data from the children of the original study members. They reported that the mother–offspring correlation of IQ scores was 0.30, and that the father–offspring correlation was 0.31 (these findings are based on 2202 parent–offspring pairs). Thus, there was no larger correlation for mothers than for fathers, which suggests that the similarity/dissimilarity of mtDNA does not affect the similarity/dissimilarity of IQ scores.

A tentative explanation of this null finding may be that there are no main effects of mtDNA variations on mitochondrial functioning, but that the latter is affected by mito-nuclear interactions only. However, this argument is at odds with the evidence that differences in mtDNA do have sizable effects on mitochondrial and body system functioning, as noted above. Another tentative explanation of this null finding may be that differences in mitochondrial functioning translate only with a very small effect size into differences in intelligence—too small to be detected in the study of Whitley et al. (2011). In this case, there is a place for many more factors that contribute to the variance of intelligence, which run outside the mechanisms of mitochondrial functioning, be they biological or social in nature.

## 2. Conclusions

Taken together, Geary’s theory is very compelling because it provides an elegant account of many important empirical phenomena of intelligence research. Moreover, it integrates biological and cognitive approaches to studying intelligence by acknowledging that elementary cognitive processes underlying human intelligence are bound to the integrity of the brain, which is one of many bodily organs and is, therefore, affected by basic physiology. We discussed different findings from neurocognitive, cognitive enhancement, and behavioral genetics research that challenged various aspects of his theory. First, we discussed recent research from cognitive psychology and cognitive neuroscience that questioned Geary’s prediction that networks relying on long-distance intermodular information transmission show a greater age-related decline due to more extensive myelin degradation. As both WM and STM processes require long-distance intermodular information transmission, it is implausible to assume that individual differences in axonal integrity and neural plasticity or age-related myelin degradation account for the phenomenon of greater age-related decline in WM than in STM. Second, we looked into the nutritional interventions discussed by Geary and concluded that these nutritional supplementations only affect cognitive functioning in older adults, in individuals with certain neurodegenerative diseases, and in individuals with dietary restrictions, whereas they do not show any cognitive benefits in healthy young adults. Third, we reviewed evidence from behavioral genetics research that challenged the prediction arising from the matrilineal heredity of mitochondrial DNA that the mother–offspring correlation of intelligence should be larger than father–offspring correlation. Data from a large-scale national cohort study showed that mother– and father–offspring correlations were virtually identical.

While our reservations based on neurocognitive and cognitive enhancement research only concerned certain parts of Geary’s theory, the discussed evidence from behavioral genetics research questioned a core assumption of the theory, namely that variations in mitochondrial DNA have an effect on mitochondrial functioning, which, in turn, has an effect on human intelligence. While we cannot and do not want to rule out that some amount of variation in human intelligence can be attributed to individual differences in mitochondrial functioning, the findings discussed above let us conclude that there are likely many more factors contributing to individual differences in intelligence, ranging from genes (e.g., genome-wide polygenic scores explain up to 10% of variance in intelligence; (Plomin and von Stumm 2018)) to structural (e.g., white-matter tract integrity in the forceps minor, the corticospinal tract, the anterior thalamic radiation, the right superior longitudinal fasciculus, the uncinate fasciculus, the rostrolateral prefrontal cortex, and the inferior parietal lobe; (Booth et al. 2013; Pineda-Pardo et al. 2016; Kievit et al. 2016; Tamnes et al. 2010; Wendelken et al. 2017)) and functional brain characteristics (e.g., activation of fronto-parietal brain networks and functional connectivity related to higher-order cognitive processes; (Basten et al. 2015; Jung and Haier 2007; Hilger et al. 2017; Schubert et al. 2020)), mediating cognitive processes (e.g., processing speed, attentional control, working memory; (Engle 2018; Kovacs and Conway 2016; Schubert and Frischkorn 2020)), environmental influences (e.g., prenatally available polyunsaturated fatty acids; (Cohen et al. 2005; Lassek and Gaulin 2008)), and developmental interdependencies (Van Der Maas et al. 2006).

## Footnotes

1 We thank an anonymous reviewer for pointing out that recent evidence suggests that this association may reverse in highly intelligent individuals (i.e., those within the upper 2% of the general population), who may be at a higher risk than the general population to develop certain psychological disorders as well as physiological conditions (Karpinski et al. 2018).
